# Equivocal evidence for a link between megalencephaly-related genes and primate brain size evolution

**DOI:** 10.1038/s41598-022-12953-4

**Published:** 2022-06-28

**Authors:** Alex R. DeCasien, Amber E. Trujillo, Mareike C. Janiak, Etta P. Harshaw, Zosia N. Caes, Gabriela A. Galindo, Rachel M. Petersen, James P. Higham

**Affiliations:** 1 Department of Anthropology, New York University, New York, USA; 2 New York Consortium in Evolutionary Primatology (NYCEP), New York, USA; 3 Section on Developmental Neurogenomics, National Institute of Mental Health (NIMH), Bethesda, USA; 4 School of Science, Engineering and Environment, University of Salford, Salford, UK; 5 Department of Anthropology, Rutgers University, New Brunswick, USA; 6 Department of Art History, University of Southern California, Los Angeles, USA; 7 Eleanor Roosevelt High School, New York, USA; 8 Department of Chemistry, Yale University, New Haven, USA; 9 Columbia Secondary School for Math, Science, and Engineering, New York, USA; 10 Department of Biological Sciences, Vanderbilt University, Nashville, USA

**Keywords:** Evolutionary genetics, Molecular evolution

## Abstract

A large brain is a defining feature of modern humans, and much work has been dedicated to exploring the molecular underpinnings of this trait. Although numerous studies have focused on genes associated with human microcephaly, no studies have explicitly focused on genes associated with megalencephaly. Here, we investigate 16 candidate genes that have been linked to megalencephaly to determine if: (1) megalencephaly-associated genes evolved under positive selection across primates; and (2) selection pressure on megalencephaly-associated genes is linked to primate brain size. We found evidence for positive selection for only one gene, *OFD1*, with 1.8% of the sites estimated to have d*N*/d*S* values greater than 1; however, we did not detect a relationship between selection pressure on this gene and brain size across species, suggesting that selection for changes to non-brain size traits drove evolutionary changes to this gene. In fact, our primary analyses did not identify significant associations between selection pressure and brain size for any candidate genes. While we did detect positive associations for two genes (*GPC3* and *TBC1D7*) when two phyletic dwarfs (i.e., species that underwent recent evolutionary decreases in brain size) were excluded, these associations did not withstand FDR correction. Overall, these results suggest that sequence alterations to megalencephaly-associated genes may have played little to no role in primate brain size evolution, possibly due to the highly pleiotropic effects of these genes. Future comparative studies of gene expression levels may provide further insights. This study enhances our understanding of the genetic underpinnings of brain size evolution in primates and identifies candidate genes that merit further exploration.

## Introduction

One of the most striking aspects of humans is our large brains. Human brains are about three times larger than those of chimpanzees, bonobos, and gorillas, which are our closest living relatives and the species with the next largest brains among extant primates^1^. Both absolute and relative brain mass generally increased throughout primate evolution, with independent increases occurring in all major clades^2,3^. Explorations of the genetic underpinnings of primate brain expansions can shed light on the mechanisms by which they have occurred. While some studies have focused on identifying ‘human-specific’ genomic alterations, often by comparing humans to the other apes or to chimpanzees and rhesus macaques only (e.g., segmental duplications of *SRGAP2C* and *ARGHAP11B*^4^; human-accelerated regulatory enhancer (*HARE5*) of *FZD8*^5^), broader phylogenetic analyses have the potential to inform us about the extent to which certain genomic or neurodevelopmental processes are conserved across large-brained primate species. Previous work has typically focused on genes that regulate the extent or duration of progenitor cell production or death during neurogenesis^6–9^, and this approach has identified multiple genes and pathways that seem to have played a role in primate brain size evolution. For example, *NIN*, a gene involved in regulating neurogenic divisions of radial glial cells, was subject to positive selection during anthropoid primate evolution, and selection on this gene, as measured by the ratio of the rates of nonsynonymous to synonymous substitutions since the last common ancestor of the species in the data set (i.e., root-to-tip d*N*/d*S*), predicts brain size across anthropoid species^10^. Similarly, a recent genome-wide comparative analysis identified numerous conserved gene-brain associations (i.e., positive correlations between selection pressure and absolute or relative brain size) across three independent episodes of primate brain size expansion^11^. Additional studies have demonstrated associations between genes and the expansion of specific brain regions. For example, selection pressure on multiple genes involved in cerebellar development predicts cerebellum size across anthropoid species^12^.

Furthermore, selection on genes associated with human disorders that produce abnormal brain sizes has been proposed to play a major role in primate brain evolution^13–15^. For example, genes known to be involved in human primary autosomal recessive microcephaly have received considerable attention in the literature. These include *ASPM*, *MCPH1*, *CDK5RAP2*, and *CENPJ*, all of which are expressed in the fetal brain during neurogenesis^16–21^. These genes are involved in apoptosis^22^ and centrosome and microtubule formation^23–26^, the latter of which may regulate neural progenitor proliferation since spindle orientation influences cell division^16^ and cell cycle progression^27^. Phenotypic correlation studies in humans suggest that these genes affect total brain size and cortical surface area, but not cortical thickness, which is likely to reflect their involvement in regulating the size of the neural progenitor pool^28–30^. Previous work has demonstrated that these genes have been subject to positive selection across primates^31–34^, and that selection pressure on these genes is associated with both brain mass increases^33^ and decreases^35^ across primates (in addition to eutherian mammals more generally^36^). There is also evidence that these genes were involved in the evolution of the large human brain, as they may have been under selection in humans during the last 40 kya^37–39^ and human-specific SNPs and methylation patterns in these genes have been identified^40^. Selection on genes associated with microcephaly may also have contributed to differences in sexual dimorphism in brain size across species^41^. Consistent with this, microcephaly-related gene mutations may have sex-specific effects on human cranial volume^30^ via estrogen-mediated regulation^42^.

The many findings linking selection on microcephaly-associated genes to inter-specific variation in brain size is a validation of this candidate gene approach to investigating primate brain evolution. It also suggests that the investigation of genes associated with additional human disorders linked to abnormal brain sizes may also provide insight into the mechanisms underlying primate brain size evolution. Here, we examine 16 genes that have been linked to megalencephaly in humans, defined as an oversized brain that exceeds the age-related mean by two or more standard deviations^43^. Recent work suggests that children with both megalencephaly and autism exhibit increased cortical surface area, but not cortical thickness, relative to those who have autism only (i.e., without megalencephaly) or are developing along a typical trajectory^44^. This suggests that, similar to microcephaly-related genes, megalencephaly-related genes may affect the size of the neural progenitor pool. Dysregulation of two functionally related cellular pathways, the Ras/mitogen-activated protein kinase (RAS-MAPK) pathway and the phosphatidyl-inositiol 3-kinase/protein kinase B/mammalian target of rapamysin (PI3K-AKT-mTOR) pathway, account for the majority of megalencephaly syndromes. Both pathways are associated with cellular functions that are critical for proper brain development, including cellular proliferation, differentiation, cell cycle regulation, and survival/apoptosis. This creates the potential for these genes, like those associated with microcephaly conditions, to be involved in the genetic mechanisms underlying inter-specific variation in primate brain size. To assess the evidence for this, we examined genes in both pathways, including *PTEN*, *PIK3CA, AKT1, AKT3, STRADA, TBC1D7, CCND2,* and *MTOR* in the PI3K-AKT-mTOR pathway, and *SPRED1* and *RIN2* in the RAS-MAPK pathway^43,45^. We also examined additional genes that have been associated with megalencephaly syndromes, including *KIF7* and *OFD1* (involved in centrosome and microtubule assembly^43^), *BRWD3* (involved in the Janus kinase/signal transducers and activators of transcription signaling (JAK-STAT) pathway, activation of which stimulates cell proliferation, differentiation, migration, and apoptosis^45,46^), *GPC3* (involved in Notch signaling^45^), *EXT2* (involved in neuron elongation^47^); and *HEPACAM* (involved in cell adhesion/morphogenesis^43^). All of these genes are expressed in the fetal brain during neurogenesis (brainspan.org).

We investigated the molecular evolution of these genes in relation to brain size across primates, with two aims: **Aim 1**: To determine whether megalencephaly-associated genes evolved under positive selection across primates. Given that several microcephaly-related genes were subject to positive selection throughout primate evolution, we predicted that one or more megalencephaly-related genes would similarly be under positive selection (Prediction 1). **Aim 2**: To determine whether selection pressure on megalencephaly-associated genes is linked to measures of primate brain size. Given that these genes are involved in the proliferation and survival of neurons, that absolute brain size increases linearly with the total number of neurons^48^, and that brain and body size may be genetically and developmentally decoupled^2,49^, we predicted that selection pressure on one or more megalencephaly-related genes would be correlated with absolute (Prediction 2), but not relative (Prediction 3), brain size across primates. These predictions are in line with previous work on genes associated with microcephaly^33^.

## Results

### Aim 1: To determine whether megalencephaly-associated genes evolved under positive selection across primates

To detect positive selection across primates, we employed site models in PAML^50^, which allow d*N*/d*S* ratios to vary across sites but not across lineages^51^. We then compared the likelihoods of two types of models, including: (1) a “nearly neutral” model which allows sites to fall into two categories, representing purifying selection and neutral evolution; and (2) a “positive selection” model which allows sites to fall into three categories, including purifying selection, neutral evolution, and positive selection^52^. We did not find evidence for positive selection for most (15/16) of the genes analyzed here. In each of these cases, the likelihood of the model that allowed sites to evolve by positive selection was not significantly higher than the likelihood of the model that allowed sites to evolve by purifying or neutral selection only (Table 1). We did find evidence for positive selection for one gene, *OFD1* (likelihood ratio = 35.405, p = 2e−8, p-adj = 3.2e−7), with 1.8% of sites estimated to have d*N*/d*S* values greater than 1 (= 6.224). This supports our Prediction 1.

**Table 1:** Results for Aim 1: Testing whether megalencephaly-associated genes evolved under positive selection across primates.

Gene	One ratio (M0) model		Nearly neutral (M1a) model			Positive selection (M2a) model				Likelihood Ratio (M2a vs. M1a)	P-value	P-adj
	d*N*/d*S*	Likelihood	Proportion of sites (d*N*/d*S*)		Likelihood	Proportion of sites (d*N*/d*S*)			Likelihood			
			d*N*/d*S* < 1	d*N*/d*S* = 1		d*N*/d*S* < 1	d*N*/d*S* = 1	d*N*/d*S* > 1				
*AKT1*	0.007	− 3625.303	0.999 (0.006)	0.001 (1)	− 3625.094	1.000 (0.006)	0.000 (1)	0 (NA)	− 3625.303	0.000	1.000	1.000
*AKT3*	0.021	− 2925.293	0.987 (0.013)	0.013 (1)	− 2923.472	0.987 (0.013)	0.013 (1)	0 (NA)	− 2923.472	0.000	1.000	1.000
*BRWD3*	0.074	− 12,041.425	0.965 (0.046)	0.035 (1)	− 12,017.838	0.965 (0.046)	0.035 (1)	0 (NA)	− 12,017.838	0.000	1.000	1.000
*CCND2*	0.024	− 2207.414	0.999 (0.023)	0.001 (1)	− 2207.403	1.000 (0.023)	0.000 (1)	0 (NA)	− 2207.414	0.000	1.000	1.000
*EXT2*	0.062	− 5732.827	0.962 (0.031)	0.038 (1)	− 5704.928	0.964 (0.032)	0.000 (1)	0.036 (1.062)	− 5704.913	0.030	0.985	0.985
*GPC3*	0.118	− 4135.848	0.864 (0.007)	0.136 (1)	− 4113.963	0.864 (0.007)	0.136 (1)	0 (NA)	− 4113.963	0.000	1.000	1.000
*HEPACAM*	0.080	− 3513.975	0.968 (0.060)	0.032 (1)	− 3507.550	0.968 (0.060)	0.032 (1)	0 (NA)	− 3507.550	0.000	1.000	1.000
*KIF7*	0.087	− 13,051.813	0.922 (0.050)	0.078 (1)	− 12,950.080	0.922 (0.050)	0.078 (1)	0 (NA)	− 12,950.080	0.000	1.000	1.000
*MTOR*	0.013	− 18,125.185	0.994 (0.008)	0.006 (1)	− 18,106.353	0.994 (0.008)	0.006 (1)	0 (NA)	− 18,106.350	0.006	1.000	1.000
*OFD1*	0.572	− 9998.686	0.538 (0.162)	0.462 (1)	− 9941.731	0.530 (0.180)	0.452 (1)	0.018 (6.224)	− 9924.029	35.405	2e−8	3.2e−7
*PIK3CA*	0.030	− 6964.649	0.973 (0.009)	0.027 (1)	− 6946.226	0.973 (0.009)	0.027 (1)	0 (NA)	− 6946.230	− 0.009	1.000	1.000
*PTEN*	0.070	− 2210.268	0.953 (0.029)	0.047 (1)	− 2208.260	0.953 (0.029)	0.047 (1)	0 (NA)	− 2208.270	0.000	1.000	1.000
*RIN2*	0.095	− 7253.544	0.949 (0.062)	0.051 (1)	− 7238.230	0.949 (0.062)	0.051 (1)	0 (NA)	− 7238.230	0.000	1.000	1.000
*SPRED1*	0.120	− 2754.241	0.954 (0.083)	0.046(1)	− 2753.040	0.954 (0.083)	0.046(1)	0 (NA)	− 2753.040	0.000	1.000	1.000
*STRADA*	0.114	− 3582.065	0.943 (0.080)	0.057 (1)	− 3572.440	0.943 (0.080)	0.057 (1)	0 (NA)	− 3572.440	0.000	1.000	1.000
*TBC1D7*	0.139	− 2518.094	0.928 (0.083)	0.072 (1)	− 2512.990	0.928 (0.083)	0.072 (1)	0 (NA)	− 2512.990	0.000	1.000	1.000

For both the nearly neutral (M1a) and positive selection (M2a) models, we provide the proportion of sites falling into each selection category (purifying: d*N*/d*S* < 1; neutral: d*N*/d*S* = 1; positive: d*N*/d*S* > 1) and the estimated d*N*/d*S* values for each category. We also provide the likelihood values for each of these models, in addition to the one-rate (M0) model. Likelihood ratios were calculated using the formula:

− 2[loglikelihood(M1a) − loglikelihood(M2a)]. P-values were calculated by comparing likelihood ratios to critical values of the chi-square distribution using two degrees of freedom. P-adj = FDR corrected p-values. Significant p-values (p < 0.05) are in bold. There is evidence for positive selection for only one gene analyzed here, *OFD1*.

### Aim 2: To determine whether selection pressure on megalencephaly-associated genes is linked to measures of primate brain size

**Figure 1:**
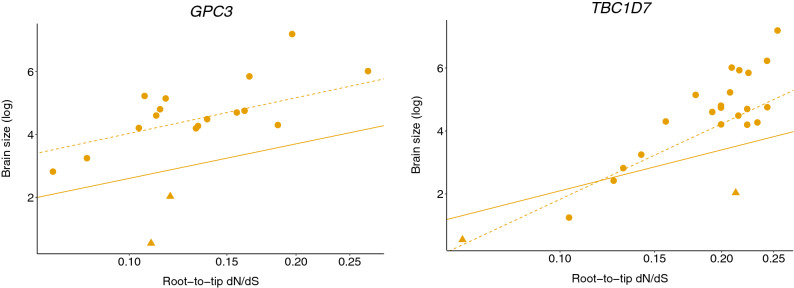
Two candidate genes exhibit associations between selection pressure and brain size in a subset of analyses. We present PGLS regression models of absolute brain size (log) ~ root-to-tip d*N*/d*S* (log) for *GPC3* (left) and *TBC1D7* (right). Each species is represented by a data point. The regression lines from models including all species are solid and those from models excluding *Callithrix jacchus* and *Microcebus murinus* are dashed. *Callithrix jacchus* and *Microcebus murinus* are represented by triangles. Regressions are significant (nominal p < 0.05) only when *Callithrix jacchus* and *Microcebus murinus* are excluded (see “Methods”, Table 2, Supplementary Table S1).

To examine relationships between selection pressure (i.e., root-to-tip d*N*/d*S*) and either absolute or relative brain size, we employed free-ratios branch models in PAML^50^, which allow d*N*/d*S* ratios to vary across branches but not across sites. We calculated root-to-tip d*N*/d*S* values for each species by the addition of d*N* values and d*S* values from the root to the terminal species branch and taking the ratio of the sums. These values were then used as predictors in phylogenetic generalized least squares (PGLS) regression models of brain size. We found no evidence for significant associations between selection pressure (i.e., root-to-tip d*N*/d*S* values) and absolute brain size for any of the genes analyzed (p-adj > 0.05; Table 2). When *Callithrix jacchus* and *Microcebus murinus* were excluded (i.e., species that have experienced secondary evolutionary decreases in brain size^3^; see “Methods”), we detected a positive relationship between selection pressure and absolute brain size for *GPC3* and *TBC1D7*; however, these associations were not significant after FDR correction (*GPC3*: estimate = 1.637, p = 0.017, p-adj = 0.119) and *TBC1D7* (estimate = 3.463, p = 0.011, p-adj = 0.119) (Fig. 1; Supplementary Table S1). The relationship for *GPC3* may be linked to increased d*N* specifically (multiple regression: d*N* estimate = 1.883, p = 0.012, p-adj = 0.168) (Fig. 1; Supplementary Table S1). These results are bolstered by sensitivity analyses (in which we removed one species at a time and re-ran the regression models). Specifically, the association between root-to-tip d*N*/d*S* and absolute brain size was only significant for *TBC1D7* when *Callithrix* was excluded (p = 0.003) and for *GPC3* when *Callithrix* (p = 0.034) or *Mandrillus* (p = 0.042) were excluded. Overall, these results provide weak support for Prediction 2, as they do not survive FDR correction and may be sensitive to species sampling. Body size significantly predicted brain size in all models of relative brain size, and we did not find evidence for any significant associations between selection pressure and relative brain size in any analysis (p > 0.05; Table 2, Supplementary Table S1), consistent with our Prediction 3. We could not test any of these relationships for two genes in our analysis, *AKT3* and *PTEN*, since most species had root-to-tip d*N*/d*S* values of zero for these genes (Supplementary Tables S2, S3).

**Table 2:** Results for Aim 2: Testing whether selection pressure on megalencephaly-associated genes is linked to measures of primate brain size.

Gene	N spp	Absolute brain size											Relative brain size										
		Lambda	d*N*/d*S*			Lambda	d*N*			d*S*			d*N*/d*S*				Lambda	d*N*			d*S*		
			Est	P-value	P-adj		Est	P-value	P-adj	Est	P-value	P-adj	Lambda	Est	P-value	P-adj		Est	P-value	P-adj	Est	P-value	P-adj
*AKT1*	20	1.000	0.008	0.942	0.989	1.000	1.320	0.143	0.532	0.527	0.164	0.328	0.430*	0.009	0.870	0.967	0.430*	− 0.019	0.952	0.996	− 0.021	0.884	0.949
*BRWD3*	21	1.000	2.716	0.211	0.739	1.000	2.581	0.239	0.669	0.678	0.879	0.879	0.376*	0.472	0.590	0.967	0.355*	0.553	0.539	0.918	− 1.246	0.432	0.830
*CCND2*	23	1.000	− 0.017	0.960	0.989	1.000	− 0.004	0.990	0.991	− 2.130	0.154	0.328	0.329*	0.127	0.304	0.960	0.296*	0.117	0.357	0.918	− 0.396	0.340	0.830
*EXT2*	23	1.000	0.354	0.753	0.989	1.000	0.408	0.723	0.991	− 1.644	0.540	0.708	0.399*	0.170	0.630	0.967	0.387*	0.198	0.594	0.918	− 0.430	0.617	0.869
*GPC3*	18	1.000	1.583	0.061	0.439	0.985	1.479	0.098	0.532	− 1.895	0.110	0.308	0.485*	0.222	0.479	0.960	0.516	0.262	0.415	0.918	0.046	0.916	0.949
*HEPACAM*	22	1.000	− 0.094	0.714	0.989	1.000	0.020	0.957	0.991	− 0.915	0.708	0.826	0.246*	− 0.102	0.480	0.960	0.238*	− 0.104	0.480	0.918	− 0.038	0.949	0.949
*KIF7*	23	1.000	− 1.633	0.304	0.851	1.000	− 1.496	0.353	0.824	− 0.530	0.866	0.879	0.450*	− 1.126	0.139	0.960	0.447*	− 1.081	0.167	0.918	0.778	0.474	0.830
*MTOR*	23	1.000	− 0.107	0.861	0.989	1.000	0.084	0.886	0.991	− 10.139	0.065	0.242	0.394*	0.034	0.898	0.967	0.260*	0.137	0.609	0.918	− 3.067	0.089	0.830
*OFD1*	19	1.000	1.960	0.643	0.989	0.966	1.844	0.662	0.991	− 5.266	0.305	0.474	0.496*	− 0.283	0.824	0.967	0.485*	− 0.151	0.910	0.996	0.769	0.621	0.869
*PIK3CA*	20	1.000	0.003	0.989	0.989	0.791	0.317	0.152	0.532	− 3.791	**0.002**	**0.028**	0.369*	− 0.002	0.986	0.986	0.377*	0.001	0.996	0.996	− 0.148	0.823	0.949
*RIN2*	23	1.000	0.707	0.642	0.989	1.000	0.483	0.731	0.991	− 4.111	0.069	0.242	0.392*	0.135	0.807	0.967	0.329*	0.128	0.812	0.996	− 0.712	0.328	0.830
*SPRED1*	23	1.000	0.667	0.603	0.989	1.000	0.016	0.991	0.991	− 2.445	0.256	0.448	0.273*	− 0.996	0.072	0.960	0.271*	− 0.975	0.111	0.918	1.050	0.162	0.830
*STRADA*	23	1.000	− 1.536	0.077	0.439	1.000	− 1.470	0.115	0.532	1.110	0.556	0.708	0.382*	− 0.232	0.463	0.960	0.386*	− 0.290	0.408	0.918	0.468	0.450	0.830
*TBC1D7*	23	1.000	1.880	0.094	0.439	1.000	0.938	0.431	0.862	− 5.412	**0.030**	0.210	0.345*	0.361	0.382	0.960	0.365*	0.195	0.656	0.918	− 1.621	0.204	0.830

Lambda values, coefficient estimates (for the predictors in the second row of table), and associated p values are provided for 4 models for each gene, including:

*Absolute brain size models.*

(1) log(brain size) ~ log(d*N*/d*S*).

(2) log(brain size) ~ log(d*N*) + log(d*S*).

*Relative brain size models.*

(3) log(brain size) ~ log(body size) + log(d*N*/d*S*).

(4) log(brain size) ~ log(body size) + log(d*N*) + log(d*S*).

There is no evidence of a significant association between d*N*/d*S* and either absolute or relative brain size for any gene. P-adj = FDR corrected p-values. Significant p-values (p < 0.05) are in bold. Est = coefficient estimate. N spp = number of species included in models. *Models were run using an averaged value of lambda (see “Methods”). Results did not change when lambda values of 0 or 1 were used in these models.

## Discussion

In the present study, we investigated the evolutionary histories of genes associated with human megalencephaly to examine their potential roles in primate brain size evolution. We tested whether any of the candidate genes that we selected evolved under positive selection across the primate phylogeny, and identified one gene with this pattern, namely *OFD1*; however, we did not detect a relationship between selection pressure on this gene and brain size across species. This suggests that selection for changes to other (i.e., non-brain size) phenotypes facilitated evolutionary changes to this gene. Although we did not identify any significant associations between selection pressure and brain size for any of our candidate genes, we did find positive relationships for *GPC3* and *TBC1D7* when phyletic dwarfs were excluded; however, these findings should be interpreted with caution since: (i) they did not survive FDR correction; (ii) the association for *GPC3* may be sensitive to species sampling; and (iii) increasing dN/dS values may reflect positive or relaxed selection. Accordingly, our results represent equivocal evidence that some megalencephaly-associated genes may have been involved in primate brain size evolution.

Although we found that one of our candidate genes, *OFD1*, evolved under positive selection across primates, selection pressure on this gene does not predict brain size. Accordingly, it is likely that selection for changes to other, non-brain size phenotypes influenced by this gene drove evolutionary changes to its coding regions. While certain mutations to this gene cause syndromes that include megalencephaly and central nervous system deficits, reflecting its role in brain development, *OFD1* is also involved in the growth and development of other parts of the body, including limb bud patterning and bone development^53^. Accordingly, selection for changes to limb morphology or proportions, which vary greatly across primates, could account for our findings. Interestingly, previous work identified signatures of positive selection on *OFD1* for certain mammalian branches, including the internal branch leading to the eutherians and the terminal branches leading to opossum, horse and tree shrew^54^. Although this study did not detect positive selection on any primate branches, this may reflect that only four primate species were included in their analysis.

We did identify two instances in which selection pressure predicted absolute brain size across species, namely for *GPC3* and *TBC1D7*, when *Callithrix jacchus* and *Microcebus murinus* were excluded, but the association did not survive FDR correction. While a trend of increasing brain size is typical of most primate lineages, previous work has suggested that both callitrichids and cheirogaleids underwent secondary reductions in brain size as a consequence of selection for decreased body size (phyletic dwarfism^3^), and that some microcephaly genes actually facilitated this decrease in callitrichids^35^. In line with this, studies of microcephaly genes suggest that callitrichids are outliers in primate-wide analyses^33^, and that, within this clade, there is a negative relationship between root-to-tip d*N*/d*S* and brain size^35^. Together with these findings, our results suggest that megalencephaly-associated genes may similarly be involved in both evolutionary increases and decreases in brain size. However, root-to-tip d*N*/d*S* values were less than 1, so we must acknowledge that increasing d*N*/d*S* values in larger-brained lineages may reflect either increasing positive selection or relaxed selection.

For *GPC3*, the detected relationship between selection pressure and absolute brain size appears to be driven by an acceleration of *dN* specifically (Supplementary Table S1), although these results may be sensitive to species sampling (see “Results”). Previous work suggests that certain alterations to this gene lead to Simpson–Golabi–Behmel syndrome, an overgrowth syndrome that is often associated with megalencephaly. Specifically, this gene regulates embryonic growth by inhibiting the Hedgehog signaling pathway through competition for the hedgehog receptor^55,56^. These pathways may directly affect brain growth since hedgehog signaling increases the proliferation of neocortical precursors^57^. Additionally, neuronal differentiation is associated with upregulation of *GPC3*, suggesting the extracellular components encoded by this gene also help regulate the distribution and activity of extracellular signaling molecules during neuronal development^58^.

For *TBC1D7*, the relationship between selection pressure and brain size may be driven by an acceleration of d*N* and/or a deceleration of *dS* (neither coefficient estimate is significant in the multiple regression model; Supplementary Table S2). Although this result is not as straightforward as that for *GPC3*, it does not necessarily represent evidence against selection. Specifically, both *dN* and *dS* depend on local mutation rates, so when *dS* decreases (thereby changing *dN/dS*), we would expect *dN* to also decrease in the absence of selection. In this case, maintaining an elevated *dN* relative to a decreasing *dS* may reflect selection on *TPC1D7* that is relevant to brain size evolution. This gene negatively regulates cell growth via suppression of mTOR (mammalian target of rapamycin) signaling^59^, and mTOR complexes regulate many functions critical to brain development, including proliferation, differentiation and migration^60^. Interestingly, a recent comparison of human organoid, chimpanzee organoid, and macaque primary cells suggested that human radial glia exhibit relatively greater increased mTOR activation^61^.

After FDR correction, we did not detect an association between selection pressure and brain size in any analysis. These findings were unexpected given the established links between primate brain size and genes associated with microcephaly, another disorder that produces abnormal brain sizes^33^. While parameter uncertainty may be due to low species sample sizes (and in some analyses, the inclusion of phyletic dwarfs), lack of significant associations may also reflect that genes associated with megalencephaly are within intermediary signaling pathways that have highly pleiotropic functions. In line with this, most syndromes that cause megalencephaly, including those linked to the genes examined here, also cause body overgrowth, cancers, and epilepsy as part of generalized overgrowth syndromes^45^. These pleiotropic, and sometimes also deleterious, effects of mutations to megalencephaly-related genes are likely to constrain their evolvability due to widespread, multivariate stabilizing selection^62^. For example, certain alterations to these genes could shift some traits (e.g., brain size) closer to their optima while simultaneously shifting other traits (e.g., body size) away from their optima, thereby hampering the ability of these genes to respond to selection for increased brain size. This is in contrast to microcephaly, which is not usually associated with profound somatic growth abnormalities^63^. Although the specific mechanisms that lead this phenotype to be brain-specific are not fully understood, new work suggests it may reflect differences between tissues in the expression of certain inhibitory splicing proteins when certain mutations create binding sites for these proteins within regions containing specific microcephaly genes^64^.

A subset of the results presented here partially overlap with prior work from Boddy et al.^11^, who took a different approach—performing a genome-wide analysis of thousands of orthologous genes across three independent episodes of brain size increase in primates. Many of the genes examined here were not included in the prior study (i.e., *PTEN*, *PIK3CA, AKT3, MTOR, RIN2, EZH2*, *MED12, OFD1*, *BRWD3*, *GPC3*) due to their justifiably conservative filtering approach. However, some genes did overlap, and neither study found significant associations between brain size and selection pressure for *AKT1, STRADA, CCND2,* or *SPRED1.* For *KIF7,* Boddy and colleagues^11^ found that the d*N*/d*S* ratio was higher in the *Colobus* versus *Papio* lineages, even though the latter exhibit larger brains, in line with our finding of a (non-significant) negative association between selection pressure on this gene and brain size. Finally, Boddy and colleagues^11^ found that selection pressure on *TBC1D7* predicts a measure of relative brain size (EQ), while we found a weak association with absolute brain size; however, our analyses differ greatly with regard to species sampling (6 species versus 23 species).

The work presented here enhances our understanding of the shared genetic basis underlying changes in brain size across primates. While numerous studies have examined genes associated with human microcephaly, this is the first study, to our knowledge, to focus explicitly on genes associated with megalencephaly. While we focused on how changes to the structure of the proteins encoded by these genes may have played a role in primate brain size evolution, future studies of comparative variation in the regulation and expression levels of these genes may provide further insights. Interestingly, previous work suggests that the expression levels of multiple genes analyzed here: (1) are differentially expressed between human and rhesus macaque brains during the prenatal and early postnatal periods^65^; (2) are differentially expressed between human and chimpanzee cerebral organoids^66^; and (3) exhibit delayed patterns of change during human brain development relative to macaque brain development^67^. In addition, the expression level of *PTEN* appears to be correlated with brain size across primate species (N = 18), and there is evidence for selection in the proximal regulatory region of this gene^68^. Given that different genes in our analyses showed signatures of positive selection or equivocal gene-phenotype associations, this study highlights the importance of including phenotypic data when attempting to identify genes involved in brain size evolution. Furthermore, although the majority of neurological differences between species may be due to differences in gene expression, this work, along with studies of microcephaly-related genes, suggest that coding sequence changes also play an important role.

## Methods

### Data collection

For each candidate gene, we retrieved coding sequences from primate species that had whole genome sequences available on NCBI (maximum N = 23 species): *Callithrix jacchus*, *Saimiri boliviensis*, *Aotus nancymaae*, *Cebus capucinus imitator*, *Cercocebus atys*, *Mandrillus leucophaeus*, *Papio anubis*, *Macaca mulatta*, *Macaca nemestrina*, *Chlorocebus sabaeus*, *Nasalis larvatus*, *Rhinopithecus roxellana*, *Rhinopithecus bieti*, *Colobus angolensis*, *Gorilla gorilla gorilla*, *Pan paniscus*, *Pan troglodytes*, *Homo sapiens*, *Pongo abelii*, *Nomascus leucogenys*, *Tarsius syrichta*, *Microcebus murinus*, and *Otolemur garnettii* (GenBank accession numbers in Supplementary Tables S4, S5). Annotations of coding sequences were confirmed via BLAST searches against each species’ genome assembly, using the *Homo sapiens* exon sequences as queries. Exons missing from automated annotations were completed via these BLAST searches, where possible. We confirmed the existence of or filled gaps in the coding regions of the candidate genes by conducting BLAST searches against the Short Read Archive (SRA). Exon sequences were aligned in Geneious^69^ using MUSCLE and default settings (alignments are available in Supplementary Data). Sequences that aligned poorly were removed from alignments and excluded from downstream analyses. Missing alignment data were excluded from PAML analyses (cleandata = 1)^50^. Accordingly, to preserve robust sampling across each gene sequence, species with < 70% gene coverage were excluded, and species sample sizes varied slightly across genes (*AKT1*: N = 20; *AKT3*: N = 23; *BRWD3*: N = 21; *CCND2*: N = 23; *EXT2*: N = 23; *GPC3*: N = 18; *HEPACAM*: N = 22; *KIF7*: N = 23; *MTOR*: N = 23; *OFD1*: N = 19; *PIK3CA*: N = 20; PTEN: N = 23; *RIN2*: N = 23; *SPRED1*: N = 23; *STRADA*: N = 23; *TBC1D7*: N = 23). Brain and body size data were collected for these species from published literature sources^3,70–72^ (see Supplementary Table S6).

### Statistical analyses

A common measure used to infer selection pressures acting on coding regions of genes is the ratio of the rates of nonsynonymous to synonymous base changes (d*N*/d*S*). Nonsynonymous changes refer to those that result in an amino acid change, while synonymous mutations refer to those that do not cause a change in the amino acid sequence.

### Aim 1: To determine whether megalencephaly-associated genes evolved under positive selection across primates

To detect positive selection across primates, we employed site models in PAML (version 4.8)^50^, which allow d*N*/d*S* ratios to vary across sites but not across lineages^51^. We compared two types of models, including: (1) a “nearly neutral” model (denoted as ‘M1a’ in PAML) which allows sites to fall into two categories, representing purifying selection (d*N*/d*S* < 1) and neutral evolution (d*N*/d*S* = 1); and (2) a “positive selection” model (denoted as ‘M2a’ in PAML) which allows sites to fall into three categories, including purifying selection (d*N*/d*S* < 1), neutral evolution (d*N*/d*S* = 1), and positive selection (d*N*/d*S* > 1)^52^. These nested models were compared using the likelihood ratio test statistic − 2[loglikelihood(M1a) − loglikelihood(M2a)], with degrees of freedom equal to the difference in the number of parameters estimated by each model. When model M2a exhibited a significantly higher likelihood value than model M1a, this was taken as evidence for positive selection. Small negative likelihood ratio values were assumed to be estimates of zero^73^.

### Aim 2: To determine whether selection pressure on megalencephaly-associated genes is linked to measures of primate brain size

To examine relationships between selection pressure (i.e., root-to-tip d*N*/d*S*) and either absolute or relative brain size, we followed other studies^74–78^ in employing free-ratios branch models in PAML (version 4.8)^50^, which allow d*N*/d*S* ratios to vary across branches but not across sites. We then calculated root-to-tip d*N*/d*S* values for each species by the addition of d*N* values and d*S* values from the root to the terminal species branch and taking the ratio of the sums. These values were set as species data and used as predictors in regression models of brain size. Although some previous studies that used this approach incorporated the d*N*/d*S* values of terminal branches^79,80^, the root-to-tip d*N*/d*S* is a more appropriate measure because it is more inclusive of the evolutionary history of a locus. In addition, some studies have used lineage-specific branch models (i.e., two-branch models), in which one model is run for each lineage with d*N*/d*S* constrained to one value over all branches leading to that lineage, to obtain root-to-tip d*N*/d*S* values for each species^11,33^. The approach used here (and in other studies^74–78^) may produce root-to-tip d*N*/d*S* values that are more comparable across species, since species with shared internal branches will have the same values for those branches included in their root-to-tip d*N*/d*S* calculations; however, we acknowledge that the free ratio model implemented here is relatively more parameter rich than the two-branch model, which may impact parameter estimation. In any case, the results of both methods are expected to be similar since they both rely on reconstructed nucleotide sequences and neither is able to address the uncertainty surrounding rate estimates. For models of absolute brain size, we modelled brain size as a function of root-to-tip d*N*/d*S*. We modeled relative brain size by also including body size as a predictor. Additionally, we performed multiple regressions for both relative and absolute brain size models in order to examine relationships with d*N* and d*S* as independent variables. Prior to analysis, all variables were log-transformed to ensure that residuals were normally distributed. We report nominal and FDR adjusted p-values^81^.

Species do not represent independent data points due to their shared evolutionary history, such that closely related species are more likely to exhibit similar phenotypes to each other than are distantly related species. To control for shared evolutionary history, we used phylogenetic generalized least squares (PGLS) regression models, incorporating the topologies and branch lengths from the GenBank taxonomy consensus tree provided on the 10kTrees website^82^. For each model, we allowed the phylogenetic scaling factor (λ) to take the value of its maximum likelihood. In some of the analyses, maximum-likelihood estimations of λ were equal to zero, and the log-likelihood plots of lambda were very flat (i.e., all values of lambda had very similar likelihood values, suggesting uncertainty regarding the best value of lambda), which may reflect low species sample sizes. Accordingly, these models were run using an average value of lambda, weighted according to likelihood (although results did not change when lambda values of 0 were used for these models). We also repeated these analyses with lambda = 0 and 1.

Previous work has suggested that callitrichids and cheirogaleids underwent recent evolutionary decreases in brain size^3^ and that some microcephaly genes actually facilitated this decrease in callitrichids, altering the expected relationship between root-to-tip d*N*/d*S* and brain size in these species^35^. Accordingly, we repeated our regression analyses excluding both *Callithrix jacchus* and *Microcebus murinus*. To test the sensitivity of these results, we re-ran relevant models removing one species at a time.

## Supplementary Information

Supplementary Information 1.

Supplementary Information 2.

Supplementary Information 3.

Supplementary Information 4.

Supplementary Information 5.

Supplementary Information 6.

Supplementary Information 7.

Supplementary Information 8.

Supplementary Information 9.

Supplementary Information 10.

Supplementary Information 11.

Supplementary Information 12.

Supplementary Information 13.

Supplementary Information 14.

Supplementary Information 15.

Supplementary Information 16.

Supplementary Information 17.

## Data Availability

GenBank accession numbers for all coding sequences retrieved and aligned for the current study are listed in Supplementary Tables S4, S5. All alignments generated and analyzed are available as supplementary material (as .phy files).
