# Supplementary material for: Equivocal evidence for a link between megalencephaly-related genes and primate brain size evolution
Source: Sci Rep. 2022 Jun 28;12:10902. doi: 10.1038/s41598-022-12953-4 (PMC9239989; doi:10.1038/s41598-022-12953-4)
Supplement: Supplementary file 5 — Supplementary Information 5. [file 41598_2022_12953_MOESM5_ESM.pdf]

Supplementary Materials for 'Equivocal evidence for a link between megalencephaly-related genes and primate brain size evolution'

Alex R. DeCasien[1,2,3]*, Amber E. Trujillo[1,2], Mareike C. Janiak[4,5] , Etta P. Harshaw[6,7], Zosia N. Caes[8,9], Gabriela A. Galindo[1], Rachel M. Petersen[1,2,10], James P. Higham[1,2]

1. Department of Anthropology, New York University, New York, USA
2. New York Consortium in Evolutionary Primatology (NYCEP), New York, USA
3. Section on Developmental Neurogenomics, National Institute of Mental Health (NIMH), Bethesda, USA
4. School of Science, Engineering & Environment, University of Salford, Salford, UK
5. Department of Anthropology, Rutgers University, New Brunswick, USA
6. Department of Art History, University of Southern California, Los Angeles, USA
7. Eleanor Roosevelt High School, New York, USA
8. Department of Chemistry, Yale University, New Haven, USA
9. Columbia Secondary School for Math, Science, and Engineering, New York, USA
10. Department of Biological Sciences, Vanderbilt University, Nashville, USA

*Correspondence: alex.decasien@gmail.com (A.R. DeCasien)

Supplementary Data Guide

Supplementary Table S1. Additional results for Aim 2: Testing whether selection pressure on megalencephaly-associated genes is lined to measures of primate brain size when *Callithrix* and *Microcebus* are excluded

Lambda values, coefficient estimates (for the predictors in the third row of table), and associated p-values are provided for 4 models for each gene, including:

Absolute brain size models

1) log(brain size) ~ log(dN/dS)

2) log(brain size) ~ log(dN) + log(dS)

Relative brain size models

3) log(brain size) ~ log(body size) + log(dN/dS)

4) log(brain size) ~ log(body size) + log(d*N*) + log(d*S*)

There is of a significant association between d*N*/d*S* and absolute for *GPC3* and *TBC1D7*. Significant p-values (p<0.05) are in bold. Est = coefficient estimate; N spp = number of species included in models. *Models were run with lambda = 1 since log-likelihood plots of lambda were very flat.

Supplementary Table S2. Root-to-tip d*N*/d*S*, d*N*, and d*S* values for candidate genes 1-8

This table contains root-to-tip d*N*/d*S*, d*N*, and d*S* values for 8 of the 16 candidate genes: *AKT1, AKT3, BRWD3, CCND2, EXT2, GPC3, HEPACAME, KIF7*. These values were obtained from PAML branch models, provided in the methods section. Gray boxes containing 'NA' values indicate instances in which exon sequences could not be aligned for the respective species (due to extensive gaps in that area of the genome).

Supplementary Table S3. Root-to-tip d*N*/d*S*, d*N*, and d*S* values for candidate genes 9-16

This table contains root-to-tip d*N*/d*S*, d*N*, and d*S* values for 8 of the 16 candidate genes: *MTOR, OFD1, PIK3CA, PTEN, RIN2, SPRED1, STRADA, TBC1D7*. These values were obtained from PAML branch models, provided in the methods section. Gray boxes containing 'NA' values indicate instances in which exon sequences could not be aligned for the respective species (due to extensive gaps in that area of the genome).

Supplementary Table S4. Accession numbers for candidate genes 1-8

This table contains accession numbers for sequences used in analyses for 8 of the 16 candidate genes: *AKT1, AKT3, BRWD3, CCND2, EXT2, GPC3, HEPACAME, KIF7*. Gray boxes containing 'NA' values indicate instances in which exon sequences could not be aligned for the respective species (due to extensive gaps in that area of the genome).

Supplementary Table S5. Accession numbers for candidate genes 9-16

This table contains accession numbers for sequences used in analyses for 8 of the 16 candidate genes: *MTOR, OFD1, PIK3CA, PTEN, RIN2, SPRED1, STRADA, TBC1D7*. Gray boxes containing 'NA' values indicate instances in which exon sequences could not be aligned for the respective species (due to extensive gaps in that area of the genome).

Supplementary Table S6. Brain and body mass for 23 primate species

This table contains brain and body masses are provided in grams, and sources for these data (in addition to any relevant notes) are also listed.

Supplementary Files

Each '*gene_name_seq*.phy' file contains the alignment compiled for that candidate gene using Geneious (see Methods).

Supplementary Table S1. Additional results for Aim 2: Testing whether selection pressure on megalencephaly-associated genes is linked to measures of primate brain size when Callithrix and Microcebus are excluded

		Absolute brain size											Relative brain size										
		dN/dS				dN				dS			dN/dS				dN				dS		
**Gene**	**N spp**	**Lambda**	**Est**	**P-value**	**P-adj**	**Lambda**	**Est**	**P-value**	**P-adj**	**Est**	**P-value**	**P-adj**	**Lambda**	**Est**	**P-value**	**P-adj**	**Lambda**	**Est**	**P-value**	**P-adj**	**Est**	**P-value**	**P-adj**
AKT1	19	1.000	-0.012	0.911	0.991	1.000	0.691	0.714	0.891	0.285	0.700	0.912	1.000*	0.001	0.986	0.986	1.000*	-0.659	0.563	0.800	-0.259	0.565	0.879
BRWD3	20	1.000	1.801	0.438	0.991	1.000	1.862	0.431	0.891	0.497	0.912	0.912	1.000*	0.901	0.494	0.916	1.000*	0.899	0.509	0.800	-0.935	0.718	0.905
CCND2	21	1.000	0.032	0.921	0.991	1.000	0.070	0.823	0.891	-2.055	0.185	0.518	1.000*	0.051	0.779	0.916	1.000*	0.057	0.759	0.800	-0.408	0.666	0.905
EXT2	21	1.000	0.014	0.990	0.991	1.000	0.093	0.932	0.932	-1.885	0.457	0.861	1.000*	0.172	0.777	0.916	1.000*	0.158	0.800	0.800	0.152	0.919	0.919
GPC3	16	0.799	1.637	**0.017**	0.119	0.906	1.883	**0.012**	0.168	-0.714	0.455	0.861	1.000*	0.316	0.473	0.916	0.443	0.571	0.131	0.761	0.124	0.776	0.905
HEPACAM	20	1.000	-0.098	0.686	0.991	1.000	0.082	0.827	0.891	-1.438	0.556	0.865	1.000*	0.039	0.776	0.916	1.000*	0.067	0.755	0.800	-0.281	0.841	0.906
KIF7	21	1.000	-2.206	0.149	0.522	1.000	-2.135	0.170	0.793	0.678	0.818	0.912	1.000*	-1.427	0.099	0.812	1.000*	-1.424	0.110	0.761	1.690	0.316	0.879
MTOR	21	1.000	-0.007	0.991	0.991	0.833	0.342	0.544	0.891	-12.577	**0.007**	**0.049**	1.000*	0.274	0.399	0.916	1.000*	0.316	0.349	0.800	-2.709	0.425	0.879
OFD1	17	0.981	1.558	0.704	0.991	0.958	1.614	0.700	0.891	-3.545	0.492	0.861	1.000*	-0.335	0.851	0.916	1.000*	-0.505	0.781	0.800	1.580	0.504	0.879
PIK3CA	19	1.000	-0.039	0.829	0.991	0.863	0.217	0.278	0.891	-3.664	**0.003**	0.042	1.000*	0.024	0.817	0.916	1.000*	0.050	0.669	0.800	-0.617	0.561	0.879
RIN2	21	1.000	0.611	0.706	0.991	1.000	0.528	0.723	0.891	-3.899	0.089	0.312	1.000*	0.245	0.790	0.916	1.000*	0.240	0.796	0.800	-1.087	0.462	0.879
SPRED1	21	1.000	0.529	0.664	0.991	1.000	0.495	0.726	0.891	-0.649	0.783	0.912	1.000*	-0.469	0.506	0.916	1.000*	-0.311	0.699	0.800	0.959	0.479	0.879
STRADA	21	1.000	-1.283	0.138	0.522	1.000	-0.900	0.377	0.891	-0.298	0.899	0.912	1.000*	-0.575	0.259	0.916	1.000*	-0.835	0.163	0.761	1.744	0.223	0.879
TBC1D7	21	1.000	3.462	**0.011**	0.119	1.000	3.041	0.064	0.448	-4.310	0.065	0.303	1.000*	1.414	0.116	0.812	1.000*	0.627	0.546	0.800	-2.839	**0.049**	0.686

Supplementary Table S2. Root-to-tip d*N*/d*S*, d*N*, and d*S* values for candidate genes 1-8

	AKT1			*AKT3*			*BRWD3*			*CCND2*			*EXT2*			*GPC3*			*HEPACAM*			*KIF7*		
**Taxa**	**dN/dS**	**dN**	**dS**	**dN/dS**	**dN**	**dS**	**dN/dS**	**dN**	**dS**	**dN/dS**	**dN**	**dS**	**dN/dS**	**dN**	**dS**	**dN/dS**	**dN**	**dS**	**dN/dS**	**dN**	**dS**	**dN/dS**	**dN**	**dS**
Aotus nancymaae	NA	NA	NA	0.00	0.00	0.08	0.07	0.01	0.11	0.02	0.00	0.12	0.03	0.01	0.42	0.07	0.00	0.04	0.03	0.01	0.49	0.11	0.08	0.75
Callithrix jacchus	NA	NA	NA	0.00	0.00	0.09	NA	NA	NA	0.02	0.00	0.18	0.03	0.02	0.45	0.12	0.01	0.06	0.02	0.01	0.51	0.12	0.10	0.82
Cebus capucinus	0.01	0.00	0.35	0.00	0.00	0.08	0.09	0.01	0.11	0.02	0.00	0.14	0.04	0.02	0.46	0.19	0.01	0.06	0.01	0.01	0.45	0.13	0.09	0.75
Cercocebus torquatus atys	6.67	0.02	0.00	0.00	0.00	0.05	0.07	0.01	0.10	0.01	0.00	0.14	0.05	0.02	0.39	0.11	0.01	0.05	0.05	0.03	0.49	0.12	0.07	0.60
Chlorocebus sabaeus	0.01	0.00	0.41	0.00	0.00	0.05	0.08	0.01	0.10	0.01	0.00	0.14	0.08	0.03	0.39	0.10	0.01	0.05	0.04	0.02	0.52	0.12	0.07	0.58
Colobus angolensis	0.01	0.00	0.44	0.02	0.00	0.05	0.07	0.01	0.11	0.01	0.00	0.14	0.05	0.02	0.39	0.13	0.01	0.04	0.07	0.04	0.52	0.13	0.08	0.64
Gorilla gorilla gorilla	0.01	0.00	0.42	0.00	0.00	0.06	0.07	0.01	0.10	0.03	0.01	0.18	0.05	0.02	0.41	NA	NA	NA	0.03	0.01	0.40	0.13	0.07	0.54
Homo sapiens	0.01	0.00	0.42	0.00	0.00	0.06	0.07	0.01	0.11	0.04	0.01	0.16	0.06	0.02	0.40	0.20	0.01	0.04	0.06	0.02	0.39	0.10	0.05	0.55
Macaca fascicularis	0.01	0.00	0.39	0.00	0.00	0.06	0.07	0.01	0.11	0.03	0.00	0.15	0.06	0.02	0.39	0.13	0.01	0.05	0.04	0.02	0.53	0.11	0.07	0.61
Macaca mulatta	0.01	0.00	0.39	0.00	0.00	0.06	0.07	0.01	0.10	0.03	0.00	0.15	0.06	0.02	0.40	0.14	0.01	0.04	0.04	0.02	0.50	0.11	0.07	0.63
Macaca nemestrina	0.01	0.00	0.40	0.00	0.00	0.05	0.07	0.01	0.10	0.01	0.00	0.15	0.05	0.02	0.39	0.16	0.01	0.04	0.04	0.02	0.52	0.12	0.07	0.63
Mandrillus leucophaeus	NA	NA	NA	0.00	0.00	0.05	0.07	0.01	0.11	0.01	0.00	0.13	0.05	0.02	0.42	0.11	0.01	0.06	0.05	0.02	0.49	0.11	0.07	0.60
Microcebus murinus	0.00	0.00	0.53	0.00	0.00	0.06	0.06	0.01	0.10	0.02	0.00	0.19	0.04	0.02	0.48	0.11	0.01	0.13	0.09	0.04	0.38	0.09	0.06	0.69
Nomascus leucogenys	0.01	0.00	0.37	0.00	0.00	0.06	0.07	0.01	0.10	0.06	0.01	0.21	0.06	0.02	0.38	0.16	0.01	0.03	0.04	0.02	0.39	0.12	0.07	0.56
Otolemur garnettii	0.01	0.01	0.56	0.01	0.00	0.12	0.08	0.01	0.13	0.01	0.00	0.29	0.03	0.02	0.60	NA	NA	NA	NA	NA	NA	0.12	0.12	1.00
Pan paniscus	0.01	0.00	0.41	0.00	0.00	0.06	0.07	0.01	0.10	0.04	0.01	0.16	0.06	0.02	0.38	0.16	0.01	0.04	0.18	0.08	0.44	0.11	0.06	0.54
Pan troglodytes troglodytes	0.01	0.00	0.42	0.00	0.00	0.06	0.07	0.01	0.11	0.04	0.01	0.16	0.06	0.02	0.37	NA	NA	NA	0.05	0.02	0.35	0.10	0.05	0.53
Papio anubis	0.01	0.00	0.38	0.00	0.00	0.06	0.07	0.01	0.10	0.01	0.00	0.13	0.06	0.02	0.39	0.12	0.01	0.05	0.06	0.03	0.51	0.11	0.07	0.61
Pongo abelii	0.01	0.00	0.43	0.00	0.00	0.06	0.10	0.01	0.11	0.01	0.00	0.16	0.07	0.03	0.38	0.27	0.01	0.02	0.12	0.05	0.46	0.11	0.06	0.55
Rhinopithecus bieti	0.01	0.00	0.42	0.00	0.00	0.06	NA	NA	NA	0.02	0.00	0.15	0.06	0.02	0.39	NA	NA	NA	0.05	0.03	0.56	0.14	0.09	0.62
Rhinopithecus roxellana	0.01	0.00	0.42	0.00	0.00	0.06	0.08	0.01	0.11	0.02	0.00	0.16	0.06	0.02	0.38	0.11	0.01	0.05	0.06	0.03	0.55	0.13	0.08	0.62
Saimiri boliviensis	0.01	0.00	0.43	0.00	0.00	0.08	0.08	0.01	0.12	0.02	0.00	0.15	0.04	0.02	0.45	0.08	0.00	0.06	0.04	0.02	0.56	0.11	0.09	0.80
Tarsius syrichta	0.01	0.00	0.39	0.12	0.01	0.08	0.08	0.01	0.08	0.00	0.00	0.33	0.08	0.07	0.84	NA	NA	NA	0.06	0.02	0.39	0.13	0.07	0.54

Supplementary Table S3. Root-to-tip d*N*/d*S*, d*N*, and d*S* values for candidate genes 9-16

	MTOR			*OFD1*			*PIK3CA*			*PTEN*			*RIN2*			*SPRED1*			*STRADA*			*TBC1D7*		
**Taxa**	**dN/dS**	**dN**	**dS**	**dN/dS**	**dN**	**dS**	**dN/dS**	**dN**	**dS**	**dN/dS**	**dN**	**dS**	**dN/dS**	**dN**	**dS**	**dN/dS**	**dN**	**dS**	**dN/dS**	**dN**	**dS**	**dN/dS**	**dN**	**dS**
Aotus nancymaae	0.01	0.00	0.39	0.72	0.07	0.10	0.01	0.00	0.30	0.23	0.04	0.17	0.10	0.03	0.30	0.13	0.02	0.16	0.05	0.02	0.45	0.13	0.04	0.30
Callithrix jacchus	0.01	0.00	0.41	0.64	0.07	0.11	0.00	0.00	0.25	0.00	0.00	0.17	0.09	0.03	0.33	0.11	0.02	0.22	0.06	0.03	0.54	0.21	0.07	0.32
Cebus capucinus	0.02	0.01	0.39	0.63	0.07	0.11	0.01	0.00	0.24	0.00	0.00	0.16	0.12	0.04	0.31	0.13	0.02	0.18	0.05	0.02	0.47	0.16	0.05	0.31
Cercocebus torquatus atys	0.01	0.00	0.38	0.60	0.06	0.10	0.11	0.02	0.19	0.00	0.00	0.12	0.09	0.02	0.26	0.11	0.02	0.18	0.06	0.03	0.57	0.19	0.06	0.33
Chlorocebus sabaeus	0.01	0.00	0.39	0.57	0.06	0.11	0.02	0.00	0.15	0.00	0.00	0.11	0.09	0.02	0.27	0.12	0.02	0.17	0.06	0.03	0.58	0.20	0.06	0.32
Colobus angolensis	0.01	0.00	0.39	0.66	0.06	0.10	0.01	0.00	0.16	0.00	0.00	0.15	0.10	0.03	0.26	0.14	0.02	0.16	0.06	0.03	0.56	0.23	0.07	0.31
Gorilla gorilla gorilla	0.00	0.00	0.37	NA	NA	NA	0.02	0.00	0.14	0.00	0.00	0.10	0.10	0.03	0.27	0.14	0.02	0.14	0.06	0.03	0.48	0.24	0.07	0.28
Homo sapiens	0.01	0.00	0.36	0.67	0.06	0.09	0.02	0.00	0.13	0.00	0.00	0.10	0.10	0.03	0.26	0.14	0.02	0.14	0.05	0.02	0.48	0.25	0.07	0.27
Macaca fascicularis	0.01	0.00	0.39	0.61	0.06	0.10	0.02	0.00	0.15	0.00	0.00	0.12	0.11	0.03	0.26	0.13	0.02	0.16	0.06	0.03	0.58	0.22	0.07	0.31
Macaca mulatta	0.01	0.00	0.40	0.60	0.06	0.10	NA	NA	NA	0.00	0.00	0.11	0.11	0.03	0.26	0.15	0.02	0.15	0.06	0.03	0.58	0.21	0.07	0.32
Macaca nemestrina	0.01	0.00	0.39	0.66	0.08	0.12	0.02	0.00	0.15	0.00	0.00	0.11	0.10	0.03	0.26	0.12	0.02	0.17	0.06	0.03	0.58	0.22	0.07	0.31
Mandrillus leucophaeus	0.01	0.00	0.39	0.60	0.06	0.10	0.02	0.00	0.14	0.00	0.00	0.12	0.09	0.02	0.26	0.12	0.02	0.17	0.07	0.04	0.57	0.21	0.06	0.31
Microcebus murinus	0.01	0.01	0.42	0.66	0.07	0.11	NA	NA	NA	0.04	0.00	0.10	0.16	0.07	0.45	0.15	0.03	0.18	0.14	0.05	0.36	0.07	0.02	0.37
Nomascus leucogenys	0.01	0.00	0.39	0.67	0.06	0.08	0.02	0.00	0.15	0.00	0.00	0.09	0.09	0.03	0.28	0.13	0.02	0.18	0.08	0.03	0.45	0.24	0.07	0.28
Otolemur garnettii	0.01	0.01	0.49	NA	NA	NA	0.01	0.00	0.29	0.05	0.01	0.16	0.13	0.08	0.61	0.16	0.03	0.17	0.05	0.03	0.52	0.13	0.04	0.31
Pan paniscus	0.01	0.00	0.36	0.66	0.06	0.09	0.05	0.01	0.13	0.00	0.00	0.11	0.09	0.02	0.27	0.16	0.02	0.14	0.06	0.03	0.46	0.22	0.07	0.31
Pan troglodytes troglodytes	0.01	0.00	0.36	NA	NA	NA	0.02	0.00	0.13	0.00	0.00	0.10	0.09	0.02	0.26	0.14	0.02	0.14	0.06	0.03	0.46	0.22	0.07	0.32
Papio anubis	0.01	0.00	0.39	0.60	0.06	0.10	NA	NA	NA	0.00	0.00	0.11	0.10	0.03	0.26	0.13	0.02	0.16	0.07	0.04	0.57	0.18	0.06	0.36
Pongo abelii	0.01	0.00	0.39	0.66	0.05	0.08	0.03	0.00	0.14	0.13	0.01	0.11	0.10	0.03	0.28	0.18	0.03	0.16	0.09	0.04	0.44	0.21	0.06	0.28
Rhinopithecus bieti	0.01	0.00	0.39	NA	NA	NA	0.12	0.02	0.16	0.00	0.00	0.14	0.08	0.02	0.27	0.13	0.02	0.15	0.08	0.04	0.55	0.20	0.06	0.30
Rhinopithecus roxellana	0.01	0.00	0.39	0.67	0.06	0.09	0.03	0.00	0.14	0.00	0.00	0.14	0.09	0.02	0.27	0.13	0.02	0.15	0.06	0.03	0.52	0.20	0.06	0.30
Saimiri boliviensis	0.01	0.00	0.40	0.69	0.07	0.10	0.01	0.00	0.29	0.00	0.00	0.14	0.14	0.04	0.30	0.09	0.02	0.20	0.06	0.03	0.50	0.14	0.04	0.31
Tarsius syrichta	0.01	0.00	0.47	0.56	0.06	0.10	0.00	0.00	0.25	0.00	0.00	0.16	0.09	0.06	0.67	0.12	0.01	0.12	0.19	0.28	1.45	0.10	0.03	0.33

Supplementary Table S4. Accession numbers for candidate genes 1-8

	AKT1		*AKT3*		*BRWD3*		*CCND2*		*EXT2*		*GPC3*		*HEPACAM*		*KIF7*	
**Taxa**	**Known RefSeq**	**Model RefSeq**	**Known RefSeq**	**Model RefSeq**	**Known RefSeq**	**Model RefSeq**	**Known RefSeq**	**Model RefSeq**	**Known RefSeq**	**Model RefSeq**	**Known RefSeq**	**Model RefSeq**	**Known RefSeq**	**Model RefSeq**	**Known RefSeq**	**Model RefSeq**
Aotus nancymaae	NA	NA	NW_012189090.1			XM_012455736.1		XM_012455904.2		XM_012452407.1		XM_012474320.1		XM_012470205.1	NW_018503064.1	XM_021674356.1
Callithrix jacchus	NA	NA	NC_013914.1		NA	NA		XM_009003440.2		XM_002755201.4		XM_008989909.2		XM_009007046.2	NC_013901.1	XM_017973300.1
Cebus capucinus		XM_017505398.1		XM_017531947.1		XM_017519568.1		XM_017546077.1		XM_017529398.1		XM_017511970.1		XM_017523932.1	NW_016107334.1	XM_017517518.1
Cercocebus torquatus atys		XM_012084168.1		XM_012036296.1		XM_012077287.1		XM_012054215.1		XM_012062330.1		XM_012061024.1		XM_012053250.1		XM_012074364.1
Chlorocebus sabaeus		XM_007987997.1		XM_007989955.1		XM_007992160.1		XM_007967271.1		XM_008000918.1		XM_007992737.1		XM_008021374.1		XM_007990356.1
Colobus angolensis		XM_011951392.1		XM_011955702.1		XM_011944125.1		XM_011931071.1	NW_012119710.1	XM_011963272.1		XM_011930705.1		XM_011934728.1		XM_011948350.1
Gorilla gorilla gorilla		XM_019009506.1		XM_004028679.2		XM_019019638.1		XM_004052524.2		XM_004050977.2	NA	NA		XM_004052354.2		XM_019010423.1
Homo sapiens	NM_001014432.1		NM_005465.4		NM_153252.4		NM_001759.3		NM_207122.1		NM_001164619.2		NM_152722.4		NM_198525.2	
Macaca fascicularis		XM_005562350.2		XM_005539655.2		XM_015443968.1		XM_005569850.1		XM_015434882.1		XM_005594608.2		XM_005580044.2		XM_005560478.2
Macaca mulatta	NM_001261625.1		NM_001266640.1			XM_015127729.2		XM_015150849.2		XM_015114422.1		XM_015128213.2		XM_015116033.1		XM_001094468.3
Macaca nemestrina		XM_011755020.1		XM_011729486.1		XM_024787598.1		XM_011745486.2		XM_011724183.1		XM_011741015.2		XM_011732050.1		XM_011752271.1
Mandrillus leucophaeus	NA	NA		XM_011981158.1		XM_011970792.1		XM_011996293.1		XM_012001352.1		XM_012001312.1		XM_012001705.1		XM_011986344.1
Microcebus murinus		XM_012746390.2		XM_012751177.2		XM_012790329.2		XM_012783145.2		XM_012790441.1		XM_012764774.1		XM_012752551.2		XM_012763339.2
Nomascus leucogenys	NM_001165894.1			XM_012506588.1		XM_003269007.3		XM_012502279.1		XM_003254461.2		XM_003272634.4		XM_003253336.2		XM_012507116.1
Otolemur garnettii		XM_012498084.1		XM_003798812.2		XM_003803556.3		XM_003796426.3		XM_003781248.1	NA	NA	NA	NA	NW_003852424.1	XM_003788669.2
Pan paniscus	NW_003852678.1			XM_003808002.3		XM_008978138.1		XM_003820341.3		XM_003808219.2		XM_003814501.2		XM_008973532.1		XM_008973901.2
Pan troglodytes troglodytes		XM_003806999.1		XM_016934876.1		XM_016943269.2		XM_001156857.5		XM_508383.5	NA	NA		XM_009424434.2		XM_016927405.1
Papio anubis		XM_024348956.1		XM_017958446.1		XM_017954111.2		XM_003905821.2		XM_009186257.2		XM_017954390.2		XM_003910894.3		XM_009210913.2
Pongo abelii		XM_009212375.2		XM_024234045.1	NC_012614.1			XM_002822783.3		XM_002821850.2		XM_024240565.1		XM_002822651.3		XM_009250144.1
Rhinopithecus bieti	NC_012605.1			XM_017870381.1	NA	NA		XM_017883284.1		XM_017858114.1	NA	NA		XM_017853859.1		XM_017861233.1
Rhinopithecus roxellana		XM_010376937.1		XM_010353265.1	NW_010800240.1			XM_010359145.1		XM_010378049.1		XM_030933937.1		XM_010384619.1		XM_010378635.1
Saimiri boliviensis		XM_003933348.2		XM_003935330.2		XM_003936828.2		XM_003942188.1		XM_003920016.2		XM_003931143.2		XM_010334671.1		XM_003921756.2
Tarsius syrichta	NW_007245946.1		NW_007245650.1	XM_008052375.2		XM_008069344.2		XM_008048699.1	NW_007257306.1	XM_008058453.2	NA	NA		XM_008057832.1		XM_021718842.1

Supplementary Table S5. Accession numbers for candidate genes 9-16

	MTOR		*OFD1*		*PIK3CA*		*PTEN*		*RIN2*		*SPRED1*		*STRADA*		*TBC1D7*	
**Taxa**	**Known RefSeq**	**Model RefSeq**	**Known RefSeq**	**Model RefSeq**	**Known RefSeq**	**Model RefSeq**	**Known RefSeq**	**Model RefSeq**	**Known RefSeq**	**Model RefSeq**	**Known RefSeq**	**Model RefSeq**	**Known RefSeq**	**Model RefSeq**	**Known RefSeq**	**Model RefSeq**
Aotus nancymaae		XM_012473670.1		XM_012434967.2	NW_012184291.1	XM_012472256.2		XM_012467566.1		XM_021673566.1		XM_012465033.1		XM_012443842.2		XM_012476566.1
Callithrix jacchus		XM_009000401.2		XM_017968373.1	NC_013910.1	XM_002758097.3		XM_017979117.1		XM_008995820.2		XM_002753561.4		XM_002748137.4		XM_008993951.2
Cebus capucinus	NW_016107330.1	XM_017503042.1		XM_017508823.1		XM_017506295.1		XM_017536375.1		XM_017512654.1		XM_017530903.1		XM_017549200.1		XM_017521503.1
Cercocebus torquatus atys		XM_012049371.1		XM_012035865.1	NW_012002159.1	XM_012086241.1		XM_012063164.1	NW_012007257.1			XM_012034725.1		XM_012070595.1		XM_012031317.1
Chlorocebus sabaeus		XM_007980575.1		XM_007991057.1	NC_023656.1	XM_007972038.1		XM_007963471.1		XM_007961294.1		XM_008017098.1		XM_008012140.1		XM_007973671.1
Colobus angolensis		XM_011935809.1		XM_011957843.1	NW_012118155.1	XM_011958660.1		XM_011938741.1		XM_011963061.1		XM_011932653.1		XM_011947949.1		XM_011962705.1
Gorilla gorilla gorilla	NC_018424.2	XM_019011017.1	NA	NA		XM_004038036.2		XM_019035119.1	NC_018444.2			XM_019011240.1		XM_004041159.2		XM_004043285.2
Homo sapiens	NM_004958.3		NM_001330210.1		NM_006218.3		NM_000314.6		NM_001242581.1		NM_152594.2		NM_001003786.2		NM_001143965.3	
Macaca fascicularis		XM_015442686.1		XM_005592994.2	NC_022273.1	XM_005546432.2		XM_005565882.2		XM_005568234.2		XM_005559135.2		XM_005584655.2		XM_005554023.2
Macaca mulatta		XM_015111100.1		XM_001098347.4	NA	NA	NM_001260965.1			XM_015149571.1	NM_001204360.1			XM_001116286.3		XM_015135572.1
Macaca nemestrina	NW_012015133.1			XM_024791672.1	NW_012011233.1	XM_011755149.1		XM_011740112.1		XM_011749872.1		XM_011759809.1		XM_011743578.1		XM_011742539.1
Mandrillus leucophaeus		XM_011996603.1		XM_011994830.1	NW_012102155.1	XM_011980373.1		XM_011990298.1		XM_011979525.1		XM_011989328.1		XM_011997060.1		XM_011989672.1
Microcebus murinus		XM_012791400.2		XM_012784803.2	NA	NA		XM_012762897.1	NC_033677.1			XM_012766374.2		XM_012747285.2		XM_012753510.2
Nomascus leucogenys	NC_019839.1			XM_030806885.1	NC_019826.1	XM_012510799.1		XM_003255173.3		XM_012512259.1		XM_003272817.2		XM_012497582.1		XM_003263521.3
Otolemur garnettii		XM_003793268.2	NA	NA	NW_003852473.1	XM_003794281.2		XM_003783677.2		XM_003798462.2		XM_003791698.2		XM_003786204.1		XM_003788311.2
Pan paniscus		XM_003822083.3		XM_003805694.3	NC_027871.1	XM_014344454.1	NM_001301760.1			XM_008962962.2		XM_008969805.1		XM_003811345.2		XM_003823152.2
Pan troglodytes troglodytes		XM_016953950.1	NA	NA	NC_006490.4	XM_016942318.1		XM_016918822.1		XM_009436892.2		XM_001138171.4		XM_016930624.1		XM_001169035.5
Papio anubis		XM_017960413.1		XM_003917416.4	NA	NA		XM_009214930.1		XM_017945051.1		XM_003900749.3		XM_009190986.2		XM_003897068.2
Pongo abelii	NC_012591.1			XM_024240796.1	NC_012594.1	XM_009239559.1	NM_001131483.1			XM_002830003.3		XM_003780630.1	NM_001132022.1			XM_009241431.1
Rhinopithecus bieti	NW_016843223.1	XM_017887986.1	NA	NA	NW_016803343.1	XM_017877282.1		XM_017868858.1		XM_017883330.1		XM_017868162.1		XM_017882900.1		XM_017891130.1
Rhinopithecus roxellana		XM_010353522.1		XM_010354059.2	NW_010789103.1	XM_010373943.1		XM_010379624.1	NW_010829246.1			XM_010358441.1		XM_010380047.1		XM_010357133.1
Saimiri boliviensis	NW_003943684.1			XM_003920320.2	NW_003943624.1	XM_003926921.2		XM_003922436.2		XM_010343724.1		XM_003935735.2		XM_003942437.2		XM_010338117.1
Tarsius syrichta	NW_007256301.1	XM_008069778.1		XM_008052678.1	NW_007251201.1	XM_008063853.1		XM_008071752.1	NW_007231443.1			XM_008064441.1	NW_007070541.1	XM_021707820.1		XM_008056827.1

Supplementary Table S6. Brain and body mass for 23 primate species

Taxa	Brain Mass (g)	Body Mass (g)	Source	Notes
Aotus nancymaae	16.78	869.98	DeCasien et al. 2017 [70]	Brain/body size data for A. lemurinus
Callithrix jacchus	7.64	272.71	DeCasien et al. 2017 [70]	
Cebus capucinus	73.79	2,798.11	DeCasien et al. 2017 [70]	
Cercocebus torquatus atys	99.81	7,770.62	DeCasien et al. 2017 [70]	
Chlorocebus sabaeus	67.25	4,312.00	Isler et al. 2008 [71]	Endocranial volumes multiplied by 1.036
Colobus angolensis	71.56	8,807.66	DeCasien et al. 2017 [70]	
Gorilla gorilla gorilla	507.25	121,327.00	DeCasien et al. 2017 [70]	
Homo sapiens	1,330.00	65,000.00	Montgomery et al. 2010 [3]	
Macaca fascicularis	66.53	4,644.11	DeCasien et al. 2017 [70]	
Macaca mulatta	88.94	6,471.73	DeCasien et al. 2017 [70]	
Macaca nemestrina	109.87	7,244.59	DeCasien et al. 2017 [70]	
Mandrillus leucophaeus	185.96	16,251.65	DeCasien et al. 2017 [70]	
Microcebus murinus	1.72	63.93	DeCasien et al. 2017 [70]	
Nomascus leucogenys	116.08	7,320.00	Montgomery 2011 [72]	
Otolemur garnettii	11.24	1,055.59	DeCasien et al. 2017 [70]	
Pan paniscus	346.24	37,522.48	DeCasien et al. 2017 [70]	
Pan troglodytes troglodytes	376.13	48,328.00	DeCasien et al. 2017 [70]	
Papio anubis	171.80	17,209.52	DeCasien et al. 2017 [70]	
Pongo abelii	409.52	48,223.06	DeCasien et al. 2017 [70]	
Rhinopithecus bieti	114.53	11,000.54	Montgomery 2011 [72]	
Rhinopithecus roxellana	122.00	14,750.00	Isler et al. 2008 [71]	Endocranial volumes multiplied by 1.036
Saimiri boliviensis	25.74	707.23	DeCasien et al. 2017 [70]	
Tarsius syrichta	3.48	107.54	DeCasien et al. 2017 [70]	
